# Is family integrated care in neonatal intensive care units feasible and good for preterm infants in China: study protocol for a cluster randomized controlled trial

**DOI:** 10.1186/s13063-015-1152-9

**Published:** 2016-01-13

**Authors:** Mingyan Hei, Xiangyu Gao, Xirong Gao, Shaohan Nong, Aimin Zhang, Qianshen Zhang, Shoo K. Lee

**Affiliations:** Department of Pediatrics, the Third Xiangya Hospital of Central South University, Tongzipo Road 138, Changsha, Hunan 410013 China; Department of Pediatrics, Xuzhou Affiliated Hospital of East South University, Xuzhou, Jiangsu 220028 China; Department of Neonatology, Hunan Children’s Hospital, Changsha, Hunan 410008 China; Department of Pediatrics, Guangdong General Hospital, Guangzhou, Guangdong 510080 China; Department of Neonatology, Hunan Provincial People’s Hospital, Changsha, Hunan 410007 China; Department of Neonatology, Shenzhen Maternal and Child Healthcare Hospital, Shenzhen, Guangdong 518028 China; Department of Pediatrics, Mount Sinai Hospital, University of Toronto, Toronto, Ontario M5G 1X5 Canada

**Keywords:** Cost-effectiveness, Family integrated care, Neonatal intensive care, Newborn, Preterm, Randomized controlled trial

## Abstract

**Background:**

By changing the paradigm of neonatal intensive care and integrating parents into the care team, the ‘family integrated care’ (FICare) model developed in Canada ensures that infants receive more consistent care and parents are better able to care for their infants within the neonatal intensive care unit (NICU) and at home. However, Chinese health policy dictates that parents are not allowed into the NICU during their infant’s stay, which inhibits this type of parent–infant interaction and may affect infant outcomes. This project aims to demonstrate that allowing parents to care for their newborn infants in the NICU improves the medical outcomes of infants.

**Methods/Design:**

This cluster randomized controlled trial will evaluate the feasibility and efficacy of FICare in six Chinese tertiary-level NICUs in China – three ‘intervention’ and three ‘control’ NICUs. The study steps are: (1) planning and preparation; (2) staff recruitment and training; (3) pilot study in two centers; (4) interim analysis and confirmation of sample size for main study; (5) implementation of main study; (6) data analysis and preparation and publication of study reports. The primary outcome measure is duration of hospital stay from admission to discharge. Secondary outcome measures are: (1) clinical outcomes, such as nosocomial infection, (2) weight gain, (3) breastfeeding, (4) time to full feed, and (5) maternal stress.

**Discussion:**

This study will assess the feasibility and cost-effectiveness of FICare in China. By establishing that FICare is a practical model of NICU care for stable preterm infants in China, this project will have a significant impact on health outcomes, medical practice and policy, and the cost of medical care. The approach used in this project could be transferable to many other areas of medical care, such as pediatrics, chronic care, and geriatrics. Data in this project can be used to inform health policy in NICUs across China so that parents are allowed to enter the NICU and be at their infant’s bedside during the baby’s hospitalization, and modifying the design of NICUs in China to facilitate the participation of parents in caring for their newborns.

**Trial registration:**

Chinese Clinical Trial Registry ChiCTR-TRC-14004736

## Background

Over the past decade, the percentage of neonatal intensive care unit (NICU) admissions in China accounted for by preterm infants has increased by a third from 19.7 to 26.2 % [1]. With advances in NICU medicine, more and more extremely premature infants are also being successfully rescued in China. However, although equipment and technology in Chinese NICUs has improved dramatically in recent decades, the overall model of care for NICU newborns remains unchanged. As a national policy, parents are not allowed to enter the NICU ward during the infant’s entire stay, with the care of these infants provided exclusively by medical specialists. This is in stark contrast to the regular nursery, where care is provided by the parents from birth.

The model of NICU care in China has been justified because of concerns over infection control and for convenient patient management by medical professionals. There is also a tradition of confinement of childbirth. This tradition, combined with general concerns about inadequate hygiene habits has allowed the current NICU model of care to go unchallenged, but is not based on any medical evidence. For example, the incidence of nosocomial infection in NICUs of hospitals in mainland China was reported to be 11.6 % in 2007 [2], which is similar to that of Taiwan [3] and Italy [4], but higher than that of the USA [5] and Canada [6]. Reducing nosocomial infections in neonatal intensive care is always of pivotal importance in decreasing the mortality and morbidity of critically ill neonates [7]. It is understandable that the medical care of critically ill preterm infants should be provided by medical professionals; after these infants are clinically stable, many still need to stay in the NICU for a very long time before they meet the discharge criteria. The current separation of mothers and their newborn infants during the infants’ stay in the NICU when they are stable but still require medical intervention is very unfair, on both the newborn infants and their mothers.

Dr. O’Brien and Dr. Shoo Lee in Mount Sinai Hospital, Toronto, Canada, [8] have recently published a pilot study of a novel model of NICU care called ‘family integrated care’ (FICare), which involves parents in the care of their NICU infants and lets them participate in the care team. The results showed that, compared with matched controls, implementing FICare resulted in a 25 % increase in weight gain, an 80 % increase in the rate of breastfeeding, a 25 % decrease in parental stress and a significant reduction in nosocomial infection and critical incident reports. The Canadian FICare study group has now started a cluster randomized controlled trial in 18 NICUs across Canada. Now the question is: If it works well in Canada, can we do anything like this for NICU infants in China? The project proposed here will take the principles of the FICare model and see how they can be applied to Chinese NICUs and what the outcomes are.

## Methods/Design

### Objectives of the study

The overall goal of the project is to evaluate the feasibility and impact of the FICare model in a multicenter cluster randomized controlled trial in six NICUs in China.

The objectives of the pilot study are:

1. To obtain information about what parents think will be the most difficult challenges to overcome in the study, and what doctors and nurses think will be the most difficult challenges to overcome in the study to aid in developing the program procedures, parent education material, and staff training material.
2. To recruit a medical staff member at each site to participate in leading the implementation of the main study at their site and train staff in educating parents and enabling them to participate in NICU care.
3. To get data from at least 90 infants and their parents (45 from the intervention site and 45 from the control site) to confirm or adjust the sample size calculation.
4. To assess the feasibility of the proposed procedures to help parents participate in taking care of their preterm but medically stable infants in the NICU, as measured by percentage enrollment in the pilot study, number of education sessions provided and parent attendance, amount of time parents spend in NICU, feedback from parents, and nursing staff.

### Hypotheses

The primary hypothesis is that the implementation of the FICare model for preterm infants in Chinese NICUs will result in a significant reduction in nosocomial infection and hence length of hospitalization. The secondary hypotheses are: (1) implementation of the FICare model in Chinese NICUs will improve maternal and infant outcomes including infant weight gain, maternal stress, and breastfeeding rates; (2) implementation of the FICare model in Chinese NICUs will result in decreased total medical expenses for preterm infants.

### Setting and sample

In this multicenter cluster randomized control trial, six tertiary-level NICUs in three different cities (two NICUs in each city) will be randomized to two groups: FICare or control groups, such that the three NICUs in each group are from different cities. A total of 588 eligible infants (294 in each group, and 98 in each NICU) for whom parental consent has been obtained will be enrolled (the enrollment rate will be approximately 11 to 18 %). An internal pilot study will be first conducted in two NICUs, one from the FICare group and one from the control group. In all, 90 infants (45 from each NICU) will be recruited for the pilot study. The recruitment period of the pilot study will take around 10 months. It is estimated that 540 to 640 eligible infants (the estimated enrollment rate is approximately 21 to 25 %) are admitted to attending NICUs each year.

Patient inclusion criteria are:

1. Infants are born at a gestational age of more than 28 weeks but less than 35 weeks;
2. Infants have been receiving enteral feeds for more than 24 h; and
3. Infants have vital signs that have been stable for more than 24 h. Patients must meet all three criteria.

Patient exclusion criteria are:

1. Infant requires either invasive or non-invasive ventilation support;
2. Infant requires surgical intervention;
3. Infant is receiving palliative care;
4. Parents have severe social issues or there is a language barrier;
5. Infant is likely to be discharged within 1 week;
6. Care-provider is not willing to commit to staying in the hospital for more than 6 h per day;
7. Parents refuse to give consent; or
8. Infant weighs less than 400 g at birth.

Patients, parents or care-givers meeting any one of these criteria should be excluded.

Patient discharge criteria are:

1. Daily enteral feed is more than 160 ml/(kg day) and infants tolerate enteral feeding well;
2. The infant has regained birth weight plus the body weight is more than 2 kg;
3. Vital signs are stable;
4. Neither intravenous medication nor oxygenation is needed;
5. The baby gains weight for at least three consecutive days; and
6. A car-seat test is passed if the parents drive.

Patients must meet all six criteria before being discharged home.

### Baseline assessments of joining neonatal intensive care units

Before inviting NICUs to join this study, a baseline assessment of candidate NICUs was completed. The baseline assessment was based on space size, number of beds, number of nurses and physicians, nurse-to-bed ratio, nurse-to-physician ratio, number of preterm infants admitted per year, and average total hospital stay of preterm infants. There must be no significant difference between joining NICUs. Finally, six tertiary NICUs were invited to join this study.

### Randomization

Since the intervention could not be completely blinded in this study, we choose a cluster randomized controlled trial design in which hospitals are randomized into two groups (intervention and control groups) instead of patients, to avoid contamination of patients in control groups. Since only six are NICUs participating in this study, the randomization was completed by a method of drawing lots by two third-party persons who are blinded to this project. In detail, the name of each participating hospital was written a piece of paper and put into an envelope by a person who was not aware of this project. The six envelopes were identical. Another person who was also blinded to this project randomly divided the six envelopes into two groups (the intervention group and the control group), with three NICUs in each group.

### Sample size calculation

The proposed sample size is based on the primary outcome of duration of hospital stay. From our previous clinical data and similar clinical research reports from our Canadian colleagues [9], the average duration of hospital stay is 28 days with a standard deviation of 15 days. We expect a decrease in hospital stay of 5 days (an approximate 18 % decrease) in the FICare group compared with the control group. Based on this expectation, the sample size was estimated using the method of Lake et al. [10], assuming an intra-cluster correlation coefficient of 0.01. We estimate that a total sample size of 586 (293 for each group or 98 for each hospital) will achieve a power of 80 % to detect the expected 18 % decrease in duration of hospital stay in the FICare group compared with the control group, taking into account a 10 % drop-out rate following enrollment in the study. This sample size will be re-adjusted in the interim analysis at the completion of the pilot study.

### Interventions

**Fig. 1:**
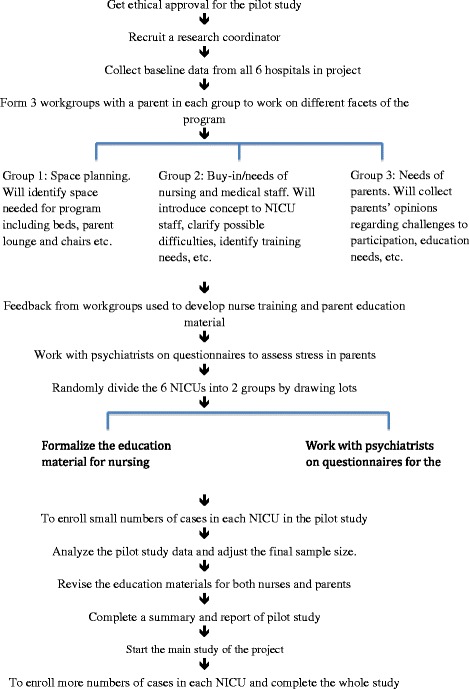
Flow chart of study design. This is a cluster randomized control trial about family integrated care (FICare) in China. This 3-year research is divided into two stages: an internal pilot study (1 year) and a main study (2 years). Ethical approval will be obtained first, followed by recruitment of research collaborator. After basement data collection, six level III NICUs in different tertiary hospitals will be randomly divided into two groups: the FICare group and the control group. Questionnaires about the demands of and attitude to FICare, and recruitment lectures about FICare will be completed in the early stage of the internal pilot study. A small number of patients will be enrolled in the internal pilot study. The data in the internal pilot study will be used to re-adjust the final sample size. Primary outcome measure data and secondary outcome measure data will be collected in both the internal pilot study and the main study. Data will be statistically analyzed at the end of the whole study

The flow chart of the study is given in Fig. 1. The program will contain the following intervention elements:

1. Parents will commit to spending at least 6 h per day in the NICU providing care for their children;
2. Parents will provide the majority of non-medical care for their infants, including feeding, bathing, dressing, changing diapers, giving oral medications, and developmental care, such as skin-to-skin care;
3. Parents will attend regular education sessions to learn the skills necessary to provide care for their infants;
4. Parents will maintain records of their infants’ progress, including temperature and weight gain, and attend rounds to discuss their infants’ care with the medical staff;
5. Nurses will work with parents to support and educate them;
6. A training program will be provided for nurses so that they are provided with the tools to support program implementation and act as coaches for parents;
7. Each NICU will provide physical supports, such as comfortable chairs at the infant’s bedside and a rest area for parents, and develop policies that support parents’ ability to spend at least 6 h in the NICU.

### Data collection procedures

A central research coordinator based at Central South University will be in charge of organizing data collection in each NICU. At least four post-graduate students will also be involved in collecting data. A research nurse at each participating NICU will be designated to work with the research coordinator and collect data. Data on maternal and infant outcome measures will be collected from patient charts using a predesigned form. The data will then be entered into a computer database along with information gathered from parents and nurses using questionnaires. Completed questionnaires will be kept in a locked cabinet at each site, accessible only by the site investigator, and the database will be password protected and accessed only by the designated research nurse at each site. The research coordinator will send the data electronically to specialists for statistical analysis every 3 months, make monthly summaries of progress and update both participating parents and the study principle investigators, organize parent activities, and obtain feedback from parents.

The clinical data to be collected are: (1) characteristics of patients: birth weight, gestational age, sex, Cesarean section, maternal gestational hypertension, maternal gestational diabetic mellitus, prolonged pre-rupture of membrane >18 h, Apgar score at 5 min after birth, SNAPPE-II (Score for Neonatal Acute Physiology–Perinatal Extension-II), antenatal steroid usage, pulmonary surfactant usage, period of intubation for invasive ventilation, period of non-invasive ventilation, period of oxygenation, age of starting enteral feeds; (2) research parameters: mortality rate, rate of breast feeding, weight gain and growth speed, time to full feeds, infection rate and infection incidence per 1000 hospitalization days, total hospitalization period, total medical hospitalization expense, extra expenses of medical staff, re-admission rate within 30 days after discharge home, parents’ questionnaire (including parental confidence in taking care of their infant and parents’ stress related to having a hospitalized infant).

The primary outcome measure is the total hospitalization period. The secondary outcome measures are: total medical expenses, infection incidence per 1000 hospitalization days, rate of breastfeeding, parental confidence and stress, assessment of feasibility and necessity of model of care, development of guidelines for supervising parents participating in the FICare model in the NICU.

The key independent variables are: rate of breast feeding, weight gain and growth speed, infection incidence per 1000 hospitalization days, total hospitalization period. The extra expenses for medical staff will include: salary for the hired research nurse, stipend for engaging doctors and nurses for extra hours of work related to the study (compared with the control groups), expenses for preparing parent education materials.

### Data analysis

Data analysis will be conducted on an intention-to-treat basis. To take into account the clustering effect, Student’s *t* test adjusted for the inflation factor (or design effect) [11, 12] will be used, to compare the primary outcome between the FICare and control groups. Furthermore, a two-level hierarchical linear regression model will be used to compare the primary outcome measure between the two groups, accounting for patient-level characteristics, such as gestational age, small for gestational age, sex, Apgar score at 5 min, maternal age, delivery mode, maternal hypertension, chorioamnionitis, maternal education, and the NICU-level covariates (NICU size, number of nurses, and neonatologists). To take into account the cluster effect, a symmetric covariance matrix structure will be used for the models.

The secondary outcome measures will also be compared between the two groups using Student’s *t* test for continuous variables and the chi-square test for categorical variables adjusted for the inflation factor. A two-level hierarchical linear regression model or a logistic regression model, as appropriate, will be applied for the comparison of the outcomes between two groups, accounting for patient-level and NICU-level characteristics.

To assess the rate of growth for the two groups, we will further examine the rate of weight change over time, if applicable. Linear or nonlinear mixed-effect models for longitudinal data will be used to compare the rate of change in weight gain between the two groups, adjusted for duration of hospital stay, and patient-level and NICU-level characteristics. An interim analysis for the internal pilot study will first be conducted using similar methods, if applicable. The preliminary results will then be used to re-justify the sample size, using the method of Lake et al. [10].

### Ethical approval and trial registration

The study received ethical approval from the ethics committee of the Third Xiangya Hospital of Central South University for Clinical Research. The ethical approval number is (2013) Ethic_CSU(S123). The study has been registered in Chinese Clinical Trial Registry (registration number, ChiCTR-TRC-14004736). Informed consent has been obtained from each participant.

In addition, the following procedures were taken to ensure compliance with ethical standards:

1. To obtain informed consent from each participating parent before enrollment;
2. To set up a system for incidence reporting, including the use of time limitations (less than 24 h) and the need for a formal written report;
3. To use patient numbers instead of patient names when summarizing data;
4. To obtain parents’ written approval for publishing their names or pictures on the research update;
5. To respect parents’ decisions to join or leave the study at any time.

### Quality control procedures

Quality control procedures include:

1. Training participating nurses in the study carefully before enrolling parents;
2. Delivering parent education lectures regularly and offering daily bedside supervision to parents;
3. Hiring a full-time research coordinator to oversee the project and research nurses at each site to collect the data;
4. Periodically distributing a research update to each participating NICU, and collecting feedback from each NICU.

In addition, the principle investigator will routinely work on the analysis of enrollment and completion numbers and feedback from parents or nurses, work with statistical specialists and discuss the progress of the study with the co-principle investigator.

## Discussion

This study is, to our knowledge, the first cluster randomized control trial to study the feasibility and cost-effectiveness of FICare in China. As in most NICUs in China, parents are not allowed to enter into the NICU ward. By establishing that FICare is a practical model of NICU care for stable preterm infants in China, this project will have a significant impact on health outcomes, medical practice and policy, and the cost of medical care.

In China, the One-Child Policy has been in place for more than three decades. Most young parents are themselves each an only child and as such may lack experience in taking care of newborn infants, which is often gained in families with multiple children through taking care of younger siblings. With changing migration patterns, new parents may also lack family support. A recent Chinese study [13] reported that mothers experience a lot of anxiety when their newborn infants are taken from them to the NICU, that breast milk secretion significantly decreases and that most parents really want more time to visit their infants. In addition, 84.2 to 96.4 % of Chinese parents wanted to learn how to take care of their newborn infants from medical professionals [14].

In Western countries, in response to this problem, many NICUs have adopted models of family-centered care in their units. This reflects a shift from the traditional focus on the biomedical aspects of an infant’s condition to a concern with seeing infants in the context of their families and recognizing the primacy of family in an infant’s life. No matter whether the infant is a preterm or a term baby. Consistent with this philosophy, many NICUs in the Western world have adopted more ‘baby-friendly’ environments and procedures, including minimizing stimulation, providing developmental care, encouraging skin-to-skin contact with parents, dimming the lights and reducing noise levels [15]. The scientific literature shows that there is both theoretical and real benefit for infants if parents are more involved in their care, especially in terms of reducing length of hospital stay [16, 17] and improving weight gain [8, 18].

China is a large country with 30 provinces, each with varying economic status, a factor that may have a significant influence on the outcomes of infants in the NICU. In addition, we do not want to conduct this study in a NICU with too many beds, as it may be difficult to maintain the quality of the study in a larger NICU, particularly in terms of problems with human resources, such as varying availability of nurses or inconsistent shift patterns. Our main objective during our baseline research was to collect data on Chinese NICUs, including the number of NICU beds, mean birth weight of patients, percentage of preterm babies, percentage of babies with gestational age <32 weeks, nurse–patient ratio, mean hospitalization period, total medical expense per patient, top three reasons for early discharge home, re-admission rate within 30 days after discharge, and possibility of allowing parents to enter NICU (0 = impossible, 1 = probable, 2 = very possible, 3 = already allowed). As a result of our analysis we identified and recruited six NICUs at the same academic level from three provinces, all from cities with an above-average economic level. The NICUs are all similar in size and have a similar patient mix. Thus, we have tried to ensure that program implementation will be as consistent as possible across the six sites with fewer barriers linked to economic status and availability of hospital and parent resources. Following success in this study we will be able to expand the program to examine whether it is feasible in regions with a lower economic status and more financial challenges.

We estimate that there will be a drop-out rate of approximately 10 % of families who enroll in the study, mostly owing to financial issues or time constraints. To minimize the effect of the biases this could introduce, we will conduct analysis with an intention-to-treat basis. There are limitations to this study. Unlike a randomized controlled trial, where patients are randomized rather than centers, some potential confounders, such as sex, gestational age, or birth weight, cannot be complete controlled for. To minimize the biases that may exist between two centers because of this, we will use multilevel models to compare the outcomes between two groups adjusting for these potential confounders in the analysis stage. We will also adjust the analysis for any potential confounders due to differences in the NICUs (NICU size, number of nurses, and neonatologists). Since the eligible infants in a NICU cannot be identified before randomization, i.e., consent cannot be obtained before randomization, this could introduce a selection bias. To minimize this bias, we will ensure that the recruitment of infants is conducted by a research coordinator who is blind to group allocation.

The project proposed here will take the principles of the FICare model as described by the Mount Sinai Hospital group and see how they can be applied to Chinese NICUs and what the outcomes are. The key pillars of this project are [8]:

1. Mothers or close family members are present in the NICU to take care of their newborn infants and collaborate with the NICU medical team in the infant’s care;
2. Parent education is provided, such that parents can truly participate;
3. Infant holding and parents being present at their infant’s bedside is supported by unit policies, physical, and environmental supports;
4. Nurses are provided with tools and education to enable families to be part of the care team; and
5. The entire care team supports the model of care.

The project is a multicenter cluster randomized controlled trial and is not a technology-based project. The approach used in this project could be transferable to many other areas of medical care, such as pediatrics, chronic care, and geriatrics. Data in this project can be used to inform health policy in NICUs across China so that parents can be allowed to enter the NICU and be at their infant’s bedsides during baby’s hospitalization, and modifying the design of NICUs in China to facilitate the participation of parents in caring for their newborn infants.

### Trial status

The study began in April, 2014, and patient enrollment commenced in July 2014. The internal pilot study was completed by the end of May, 2015. The study is in the stage of the main study. Official registration was completed in May, 2014. The registration number of the study is ChiCTR-TRC-14004736. The whole study is scheduled to be completed by the end of December, 2016.

## Abbreviations

FICare: family integrated care
NICU: neonatal intensive care unit
SNAPPE-II: Score for Neonatal Acute Physiology–Perinatal Extension-II
